# Computational investigation of stochastic Zika virus optimal control model using Legendre spectral method

**DOI:** 10.1038/s41598-024-69096-x

**Published:** 2024-08-05

**Authors:** Junjie Zhu, Feroz Khan, Sami Ullah Khan, Wojciech Sumelka, Farman U. Khan, Salman A. AlQahtani

**Affiliations:** 1 School of Mathematics, Shandong University, Jinan, 250100 China; 2 School of Mathematics and Data Sciences, Changji University, Changji, 831100 China; 3 Department of Mathematics, City University of Science and Information Technology Peshawar, Peshawar, KP 2500 Pakistan; 4 Institute of Structural Analysis, Poznan University of Technology, Piotrowo 5 Street, 60-965 Poznan, Poland; 5 Department of Mathematics, HITEC University, Taxila Cantt, Taxila, 47080 Pakistan; 6 Computer Engineering Department, College of Computer and Information Sciences, King Saud University, Riyadh, Saudi Arabia

**Keywords:** Legendre spectral collocation method, Zika virus model, Control variables, Stochastic asymptotic stability, Brownian motion, Legendre polynomials, Reproduction number, Biological techniques, Mathematics and computing

## Abstract

This study presents a computational investigation of a stochastic Zika virus along with optimal control model using the Legendre spectral collocation method (LSCM). By accumulation of stochasticity into the model through the proposed stochastic differential equations, we appropriating the random fluctuations essential in the progression and disease transmission. The stability, convergence and accuracy properties of the LSCM are conscientiously analyzed and also demonstrating its strength for solving the complex epidemiological models. Moreover, the study evaluates the various control strategies, such as treatment, prevention and treatment pesticide control, and identifies optimal combinations that the intervention costs and also minimize the proposed infection rates. The basic properties of the given model, such as the reproduction number, were determined with and without the presence of the control strategies. For $R_0<0$, the model satisfies the disease-free equilibrium, in this case the disease die out after some time, while for $R_0>1$, then endemic equilibrium is satisfied, in this case the disease spread in the population at higher scale. The fundamental findings acknowledge the significant impact of stochastic phonemes on the robustness and effectiveness of control strategies that accelerating the need for cost-effective and multi-faceted approaches. In last the results provide the valuable insights for public health department to enabling more impressive mitigation of Zika virus outbreaks and management in real-world scenarios.

## Introduction

The first incidence of the Zika virus (ZIKV) was discovered in Afria Yap, and the outbreak was initially identified in Africa^1^. Another outbreak in French Polynesia ensued after this, affecting multiple Pacific nations^2,3^, and lasting for few months. ZIKV cases were recorded in 2015 in a number of South American nations, including Brazil and Colombia^4–6^. While ZIKV is primarily transmitted through the Aedes species mosquito^7^, additionally, blood transfusions and sexual intercourse can spread it. Moreover, the dengue virus is spread by this mosquito. Other tropical areas are capable of supporting ZIKV transmission. The rash,fever and an inflated risk of neurological problems are among the symptoms and also the signs of ZIKV^8,9^. Similarly, the microcephaly may also appear in children whose mothers had ZIKV throughout in their pregnancies^10,11^. The circumstance of ZIKV in French Polynesia and Brazil has resulted in assemblage of microcephaly and some other neurological issues for which the instruction of World Health Organization (WHO) to demonstrate a Public Health Emergency of the International Concern^12,13^. Moreover, there was an epidemic in the location of French Polynesia that had been reporting of 42 precedent of Guillain-Barre syndrome between the time of the March 2014 to May 2015, where there have been 10 cases of the disease that had severe microcephaly and brain lesions^14^. However, the ZIKV has the probable to spread globally, therefore to understanding the mechanisms of the ZIKV transmission is crucial. Since it is well-known that mathematical models are applicable tools for researching, how the infectious diseases spread in the population. Mathematical models can help to regulate the threshold of reproduction number, which bring the information on how long the proposed infection finish. Numerous researchers, including those who have studied the transmission of ZIKV, have investigated mosquito-borne infections using mathematical models^15–17^. The elements influencing ZIKV’s spread can be found using mathematical models. Effective disease control is a top priority for the WHO, and using optimal time control can provide valuable theoretical insights for disease prevention and treatment. The insights gained from optimal control modeling can inform decision-making and help health care providers plan and deliver effective disease management and treatment. Optimal time control has been applied in numerous studies of mosquito-borne diseases^18,19^.

The ZIKV has unnatural significant public health challenges since its severe congenital effects, emergence and its primarily due to its rapid spread. This has constrained the development of computational and mathematical models to alleviate and understand its spread. However, the optimal control theory has been widely applied to the epidemiological models to construct the strategies for controlling such infectious diseases and its spread in the world. Such models typically consolidate various control measures such as treatment, vaccination and public health interventions to control the impact and spread of the disease.

The deterministic vs. stochastic models In the context of infectious disease modeling, both of the above models approaches have been employed in the field of epidemiology. Deterministic models use initial conditions and fixed parameters to predict the behavior of the system. In the literature there are many researcher studies the deterministic models for different diseases^20–22^. Similarly, the deterministic models with fractional derivatives were proposed by^23,24^. However, the deterministic phenomena often fail to capture the inherent randomness in the spread of the diseases, especially when the disease is at an early stage or when the number of infected individuals is small.

Stochastic models, on the other hand, incorporate random variables and processes to better represent the unpredictable nature of disease transmission^25–27^. Stochastic models are particularly useful for capturing the variability and uncertainty in the spread of diseases^28,29^. For instance, to demonstrated the stochasticity importance in the context of mosquito-borne diseases, highlighting how stochastic models can provide more realistic predictions as compared to deterministic models^30^.

In the present research study, we focus the LSCM that is one of a powerful numerical technique, that has been used to solve such optimal control problems in field of epidemiology. Moreover, the present technique also offers several advantages, including the high accuracy, fast convergence and ability to handle such complex boundary conditions.

The stochastic Optimal Control of ZIKV contributes to the existing literature by investigating a stochastic optimal control model for the ZIKV using the LSCM. This approach combines the strengths of spectral methods and stochastic modeling to address the complexities corresponding with the spread of ZIKV. Previous studies have explored various aspects of ZIKV control, including vaccination strategies^14,17^, vector control^3,4^, and public health interventions^10,11^. However, the incorporation of stochastic elements and the use of the LSCM provide a novel perspective on this problem.

In this article, we provide a novel model that use optimal control to investigate how prevention, pesticides and mass treatment impact the potential of the ZIKV to spread. We want to reduce the number of hosts and vectors that are infected with ZIKV while maximizing the effectiveness of insecticides, prevention and mass treatment.

Moreover, integration of the stochastic modeling along with the LSCM represents a significant advancement in the field of the proposed epidemiological modeling. The LSCM approach also offers the robust structure for evaluating control strategies and designing for ZIKV, computing for the uncertainties in the disease transmission. The present research work builds on the actual body of the knowledge which demonstrating the possible of the LSCM to control of infectious diseases and improve our understanding. However, the future research can further be analyze the application of the proposed methods to other infectious diseases and clarify the complex models to consolidate the additional complexities and real-world data.

## Model construction

The stochastic mathematical model in the form of ordinary differential equation has been formed in this section. Bonyah^31^ developed and examined the SIR epidemiological model, as follow:

1 $$\begin{aligned} {\left\{ \begin{array}{ll} S_H'(t)=\Lambda _H-{(1-\mu _1)\beta _1S_H(I_V+\vartheta I_H)-\mu _HS_H},\\ I_H'(t)=(1-\mu _1)\beta _1S_H(I_V+\vartheta I_H)-(\mu _H+\varsigma +\upsilon _1\mu _2)I_H,\\ R_H'(t)=(\varsigma +\upsilon _1\mu _2)I_H-\mu _HR_H,\\ S_V'(t)=\Lambda _V-(1-\mu _1)\beta _2S_VI_H-(\mu _V+\upsilon _2\mu _3)S_V,\\ I_V'(t)=(1-\mu _1)\beta _2S_VI_H-(\mu _V+\upsilon _2\mu _3)I_V. \end{array}\right. } \end{aligned}$$

Examining the stochastic phenomena of the suggested ZIKV model is the aim of our study. When opposed to the deterministic technique, the stochastic approach is thought to be more practical, particularly when modeling short-term disease data such as ZIKV and the flu. Stochastic models are better suited for precisely simulating the phenomena of interest. Hence, we have developed a stochastic system based on the equations outlined in Eq. (1):

2 $$\begin{aligned} {\left\{ \begin{array}{ll} dS_H(t)=\Lambda _H-{(1-\mu _1)\beta _1S_H(I_V+\vartheta I_H)-\mu _HS_H}+\sigma S_HdW(t),\\ dI_H(t)=(1-\mu _1)\beta _1S_H(I_V+\vartheta I_H)-(\mu _H+\varsigma +\upsilon _1\mu _2)I_H+\sigma I_HdW(t),\\ dR_H(t)=(\varsigma +\upsilon _1\mu _2)I_H-\mu _HR_H+\sigma R_HdW(t),\\ dS_V(t)=\Lambda _V-(1-\mu _1)\beta _2S_VI_H-(\mu _V+\upsilon _2\mu _3)S_V+\sigma S_VdW(t),\\ dI_V(t)=(1-\mu _1)\beta _2S_VI_H-(\mu _V+\upsilon _2\mu _3)I_V+\sigma I_VdW(t). \end{array}\right. } \end{aligned}$$

The study explores the dynamics of ZIKV transmission to humans using a susceptible; infected; recovered model. The human population at time *t*, denoted by $N_H$, is divided in this model into three categories: susceptible people $S_H(t)$, infected persons $I_H(t)$, and those who have recovered from Zika $R_H(t)$. The population vectors at time t consists of two sub-populations of mosquitoes, $N_V(t)$: susceptible mosquitoes $S_V(t)$ and infected insects $I_V(t)$. $\beta _1$ reflects the ZIKV transmission rate from mosquitoes to humans, whereas $\beta _2$ represents the transmission rate from humans to mosquitoes. Additionally, the model takes into account the following: the rate of recruitment into the susceptible vector population $\Lambda _V$, the rate of recruitment into the susceptible human population $\Lambda _H$, the rates of natural death for both hosts and vectors $\mu _H$ and $\mu _V$ respectively, the rate of recovery from treatment $\upsilon _1$, and the average infectious period for humans $\varsigma$. The research examines three limited and Lebesgue integrable control functions: pesticide control $\mu _3$, treatment control $\mu _2$, and prevention control $\mu _1$. All infectious diseases exhibit randomness in their transmission, which can be modeled by introducing white noise into deterministic system equations. In this study, we utilized this approach to obtain a stochastic counterpart of the ZIKV model in Eq. (1). In the case of the Zika virus, various factors can contribute to the variability of the source term. For instance, seasonal variations in mosquito populations, climate conditions affecting mosquito breeding and activity, human travel patterns, and effectiveness of public health interventions can all impact the rate at which new infections occur. Additionally, the variable source term may account for changes in the number of susceptible individuals due to factors like immunization campaigns, migration, or changes in human behavior in response to public health messages.

We employed the LSCM to numerically solve the ZIKV model, which has been previously used for differential and integral systems. Additionally, various authors have used this method to solve different SIR models^32–34^.

This research paper is structured as follows: in the next section, we review the Legendre polynomials and spectral method. The basic reproduction number is briefly covered in "Method Description", while "Basic Reproduction Number (R0)" presents the stability analysis. We go over the numerical results in section Stability and Convergence of the Spectral Method" and the last part discusses conclusion.

## Method description

Legendre polynomials, which have been well investigated in earlier works^35–37^, must be introduced before exploring the Legendre spectral collocation method.$Q_{n}(\xi )$ represents the $n^{th}$ order Legendre polynomial. Let us consider a function $v(\xi )$ that is defined as follows on $[-1, 1]$:

3 $$\begin{aligned} v(\xi )=\sum _{j=0}^{n}v_j Q_j(\xi ). \end{aligned}$$

The unknown Legendre coefficients are represented by $v(\xi _i)$, and the collocation points are considered to satisfy the condition $-1=\xi _0< \xi _1<...< \xi _n=1$. Where in genral:

4 $$\begin{aligned} Q_{j}(\xi )=\frac{1}{2^{j}}\sum _{i=0}^{[\frac{j}{2}]}(-1)^{i}(_{i}^{j})(_{\quad j}^{2j-2i})\xi ^{j-2i},\quad \xi \in [-1, 1] \quad (j=0,..., n ). \end{aligned}$$

$$\begin{aligned} \left[ \frac{j}{2}\right] = {\left\{ \begin{array}{ll} \frac{j}{2}, &{} \text {if }j\text { is even}, \\ \frac{j-1}{2}, &{} \text {if }j\text { is odd}. \end{array}\right. } \end{aligned}$$.

To model Eq. (2) using the ”Legendre-Gauss iteration” with a weight function, we utilized Legendre-Gauss Lobatto points $\{\tau _j\}_{j=0}^{N},$. To numerically approximate the equations, we took the integral of both sides of Eq. (2) over the interval [0, *t*], resulting in:

5 $$\begin{aligned} {\left\{ \begin{array}{ll} S_H(t)&{}=S_H(0)+\int _{0}^{t}\big (\Lambda _H-{(1-\mu _1)\beta _1S_H(u)(I_V(u)+\vartheta I_H(u))-\mu _HS_H(u)}\big )du\\ &{}+\int _{0}^{t}\sigma S_H(u)dW(u),\\ I_H(t)&{}=I_H(0)+\int _{0}^{t}\big ((1-\mu _1)\beta _1S_H(u)(I_V(u)+\vartheta I_H(u))-(\mu _H+\varsigma +\upsilon _1\mu _2)I_H(u)\big )du\\ &{}+\int _{0}^{t}\sigma I_H(s)dW(u)\\ R_H(t)&{}=R_H(0)+\int _{0}^{t}\big ((\varsigma +\upsilon _1\mu _2)I_H(u)-\mu _HR_H(u)\big )du\\ &{}+\int _{0}^{t}\sigma R_H(u)dW(u)\\ S_V(t)&{}=S_V(0)+\int _{0}^{t}\big (\Lambda _V-(1-\mu _1)\beta _2S_V(u)I_H(u)-(\mu _V+\upsilon _2\mu _3)S_V(u)\big )du\\ &{}+\int _{0}^{t}\sigma S_V(u)dW(u)\\ I_V(t)&{}=I_V(0)+\int _{0}^{t}\big ((1-\mu _1)\beta _2S_V(u)I_H(u)-(\mu _V+\upsilon _2\mu _3)I_H(u)\big )du\\ &{}+\int _{0}^{t}\sigma I_V(u)dW(u). \end{array}\right. } \end{aligned}$$

Keeping in mind the initial values $S_H(0), I_H(0), R_H(0), S_V(0)$, and $I_V(0)$ for each of the corresponding classes. We make the transformation $u=\frac{t}{2}(\tau +1)$, to explore the Legendre spectral collocation technique (LSCM) on [-1, 1]. Equation (5) may thus be expressed as follow:

6 $$\begin{aligned} S_H(t)&=S_H(0)+\frac{t}{2}\int _{-1}^{1}\bigg (\Lambda _H-(1-\mu _1)\beta _1S_H\big (\frac{t}{2}(\tau +1)\big )\big (I_V\big (\frac{t}{2}(\tau +1)\big )+ \vartheta I_H\big (\frac{t}{2}(\tau +1)\big )\big )\nonumber \\&-\mu _HS_H\big (\frac{t}{2}(\tau +1)\big )\bigg )d\tau +\frac{t}{2}\int _{-1}^{1}\sigma S_H(\frac{t}{2}(\tau +1)dW(\tau ),\nonumber \\ I_H(t)&=I_H(0)+\frac{t}{2}\int _{-1}^{1}\bigg ((1-\mu _1)\beta _1S_H(\frac{t}{2}(\tau +1)\big (I_V\big (\frac{t}{2}(\tau +1)\big )+\vartheta I_H\big (\frac{t}{2}(\tau +1)\big )\big )\nonumber \\&-(\mu _H+\varsigma +\upsilon _1\mu _2)I_H\big (\frac{t}{2}(\tau +1)\big )\bigg )d\tau +\frac{t}{2}\int _{-1}^{1}\sigma I_H\big (\frac{t}{2}(\tau +1)\big )dW(\tau ),\nonumber \\ R_H(t)&=R_H(0)+\frac{t}{2}\int _{-1}^{1}\bigg ((\varsigma +\upsilon _1\mu _2)I_H(\frac{t}{2} (\tau +1))-\mu _HR_H(\frac{t}{2}(\tau +1))\bigg )d\tau \nonumber \\&+\frac{t}{2}\int _{-1}^{1}\sigma R_H\big (\frac{t}{2}(\tau +1)\big )dW(\tau ),\nonumber \\ S_V(t)&=S_V(0)+\frac{t}{2}\int _{-1}^{1}\bigg (\Lambda _V-(1-\mu _1)\beta _2S_V(\frac{t}{2}(\tau +1))I_H(\frac{t}{2}(\tau +1)) \nonumber \\&-(\mu _V+\upsilon _2\mu _3)S_V(\frac{t}{2}(\tau +1))\bigg )d\tau +\frac{t}{2}\int _{-1}^{1} \sigma S_V\big (\frac{t}{2}(\tau +1)\big )dW(\tau ),\nonumber \\ I_V(t)&=I_V(0)+\frac{t}{2}\int _{-1}^{1}\bigg ((1-\mu _1)\beta _2S_V(\frac{t}{2}(\tau +1)I_H(\frac{t}{2}(\tau +1)) \nonumber \\&-(\mu _V+\upsilon _2\mu _3)I_H(\frac{t}{2}(\tau +1)))\bigg )d\tau +\frac{t}{2}\int _{-1}^{1} \sigma I_V\big (\frac{t}{2}(\tau +1)\big )dW(\tau ),\nonumber \\ \end{aligned}$$

next we covert this equation to semi-discretized spectral form as:

7 $$\begin{aligned} S_H(t)&=S_H(0)+\frac{t}{2}\sum _{l=0}^{N}\bigg \{\Lambda _H-\big ((1-\mu _1) \beta _1S_H\big (\frac{t}{2}(\tau _l +1)\big )\big (I_V(\frac{t}{2}(\tau _l +1)\big )\nonumber \\&+ \vartheta I_H\big (\frac{t}{2}(\tau _l +1)\big )-\mu _HS_H(s)\bigg \}\omega _l+\frac{t}{2}\sum _{l=0}^{N}\sigma S_H\big \{\frac{t}{2}(\tau _l +1)\big \}\omega _l^*,\nonumber \\ I_H(t)&=I_H(0)+\frac{t}{2}\sum _{l=0}^{N}\bigg \{(1-\mu _1)\beta _1S_H(\frac{t}{2}(\tau _l +1)(I_V(\frac{t}{2}(\tau +1))\nonumber \\&+\vartheta I_H\big (\frac{t}{2}(\tau _l +1)\big ))-(\mu _H+\varsigma +\upsilon _1\mu _2)I_H(s)\bigg \}\omega _l\nonumber \\&+\frac{t}{2}\sum _{l=0}^{N}\sigma I_H\big \{\frac{t}{2}(\tau _l +1)\big \}\omega _l^*,\nonumber \\ R_H(t)&=R_H(0)+\frac{t}{2}\sum _{l=0}^{N}\bigg \{(\varsigma +\upsilon _1\mu _2)I_H(\frac{t}{2}(\tau _l +1))-\mu _HR_H(\frac{t}{2}(\tau _l +1))\bigg \}\omega _l\nonumber \\&+\frac{t}{2}\sum _{l=0}^{N}\sigma R_H\big \{\frac{t}{2}(\tau _l +1)\big \}\omega _l^*,\nonumber \\ S_V(t)&=S_V(0)+\frac{t}{2}\sum _{l=0}^{N}\bigg \{\Lambda _V-(1-\mu _1)\beta _2S_V\big (\frac{t}{2}(\tau _l +1)) I_H(\frac{t}{2}(\tau _l+1))\nonumber \\&-(\mu _V+\upsilon _2\mu _3)S_V(\frac{t}{2}(\tau _l +1)\big )\bigg \}\omega _l +\frac{t}{2}\sum _{l=0}^{N}\sigma S_V\big \{\frac{t}{2}(\tau _l +1)\big \}\omega _l^*,\nonumber \\ I_V(t)&=I_V(0)+\frac{t}{2}\sum _{l=0}^{N}\bigg \{(1-\mu _1)\beta _2S_V(\frac{t}{2}(\tau _l +1)I_H(\frac{t}{2} (\tau _l +1)\big )\nonumber \\&-(\mu _V+\upsilon _2\mu _3)I_H(\frac{t}{2}(\tau _l +1)\big )\bigg \}\omega _l +\frac{t}{2}\sum _{l=0}^{N}\sigma I_V\big \{\frac{t}{2}(\tau _l +1)\big \}\omega _l^*,\nonumber \\ \end{aligned}$$

where the weight function for deterministic part is:

$$\begin{aligned} {\omega }_l\mathrm {=}\frac{\textrm{2}}{\mathrm {(1}-r^{\textrm{2}}_l \mathrm {)[}K^{\mathrm {'}}_{N+\textrm{1}} \mathrm {(}r_l\mathrm {)}{\mathrm {]}}^{\textrm{2}}},\ \ \ \ \ \ \ \ 0\le l\le N. \end{aligned}$$

And the weight function for stochastic part is:

$$\begin{aligned} {\omega }^{*}_{l}=\sqrt{{\omega }_{l}}\times randam {(1,}N{),}\ \ \ \ \ \ \ \ 0\le l\le N. \end{aligned}$$

Equation (5) is currently being employed to approximate $S_H(t)$, $I_H(t)$, $R_H(t)$, $S_V(t)$ and $I_V(t)$ using the Legendre polynomial.

8 $$\begin{aligned}&S_H(t)=\sum _{m=0}^{N}S_{Hm}P_m(t),\qquad I_H(t)=\sum _{m=0}^{N}I_{Hm}P_m(t),\nonumber \\&R_H(t)=\sum _{m=0}^{N}R_{Hm}P_m(t),\qquad S_V(t)=\sum _{m=0}^{N}S_{Vm}P_m(t),\nonumber \\&I_V(t)=\sum _{m=0}^{N}I_{Vm}P_m(t). \end{aligned}$$

The Legendre coefficients $S_{Hm}, I_{Hm}, R_{Hm}, S_{Vm},$ and $I_{Vm}$ correspond to the functions of each class. Utilizing the aforementioned approximation, we can simplify Eq. (7) to a more manageable form as follows:

9 $$\begin{aligned} \sum _{m=0}^{N}S_{Hm}P_m(t)&=\sum _{m=0}^{N}S_{Hm}P_m(0)+\frac{t}{2} \sum _{l=0}^{N}\bigg \{\big (\Lambda _H-(1-\mu _1)\beta _1 \sum _{m=0}^{N}S_{Hm}P_m(\eta _l)\bigg (\sum _{m=0}^{N}I_{Vm}P_m(\eta _l)\big )\nonumber \\&+\vartheta \sum _{m=0}^{N}I_{Hm}P_m(\eta _l)\bigg )-\mu _H \sum _{m=0}^{N}S_{Hm}P_m(\eta _l)\bigg \}\omega _l+\frac{t}{2}\sum _{l=0}^{N} \bigg \{\sigma \sum _{m=0}^{N}S_{Hm}P_m (\eta _l)\bigg \}\omega ^*_l,\nonumber \\ \sum _{m=0}^{N}I_{Hm}P_m(t)&=\sum _{m=0}^{N}I_{Hm}P_m(0)+\frac{t}{2} \sum _{l=0}^{N}\bigg \{(1-\mu _1)\beta _1 \sum _{n=0}^{N}S_{Hn}P_m(\eta _l)\bigg (\sum _{m=0}^{N}I_{Vm}P_m(\eta _l)\nonumber \\&+\sum _{m=0}^{N}I_{Hm}P_m(\eta _l)\bigg )-(\mu _H+\varsigma +\upsilon _1\mu _2) \sum _{m=0}^{N}I_{Hm}P_m(\eta _l)\bigg \}\omega _l\nonumber \\&+\frac{t}{2}\sum _{l=0}^{N}\bigg \{\sigma \sum _ {m=0}^{N}I_{Hm}P_m(\eta _l)\bigg \}\omega ^*_l,\nonumber \\ \sum _{m=0}^{N}R_{Hm}P_m(t)&=\sum _{m=0}^{N}R_{Hm}P_m(0)+\frac{t}{2} \sum _{l=0}^{N}\bigg \{\big (\varsigma +\upsilon _1\mu _2) \sum _{m=0}^{N}I_{Hm}P_m(\eta _l)-\upsilon _1\sum _{m=0}^{N}R_{Hm} P_m(\eta _l)\bigg \}\omega _l\nonumber \\&+\frac{t}{2}\sum _{l=0}^{N}\bigg \{\sigma \sum _{m=0}^{N}R_{Hm} P_m(\eta _l)\bigg \}\omega ^*_l,\nonumber \\ \sum _{m=0}^{N}S_{Vm}P_m(t)&=\sum _{m=0}^{N}S_{Vm}P_m(0) +\frac{t}{2}\sum _{l=0}^{N}\bigg \{\big (\Lambda _V-(1-\mu _1)\beta _2 \big (\sum _{m=0}^{N}S_{Vm}P_m(\eta _l)\big ) \big (\sum _{m=0}^{N}I_mP_m(\eta _l)\big )\nonumber \\&-(\mu _V+\upsilon _2\mu _3)\sum _{m=0}^{N} S_{Vm}P_m(\eta _l)\bigg \}\omega _l+\frac{t}{2}\sum _{l=0}^{N} \bigg \{\sigma \sum _{m=0}^{N}S_{Vm}P_m(\eta _l)\bigg \}\omega ^*_l,\nonumber \\ \sum _{m=0}^{N}I_{Vm}P_m(t)&=\sum _{m=0}^{N}I_{Vm}P_m(0)+\frac{t}{2}\sum _{l=0}^{N}\bigg \{ \big ((1-\mu _1)\beta _2 \big (\sum _{m=0}^{N}S_{Vm}P_m(\eta _l)\big ) \big (\sum _{m=0}^{N}I_{Hm}P_m(\eta _l)\big )\nonumber \\&-(\mu _V+\upsilon _2\mu _3)\sum _{m=0}^{N}I_{Hm}P_m(\eta _l)\bigg \}\omega _l +\frac{t}{2}\sum _{l=0}^{N}\bigg \{\sigma \sum _{m=0}^{N}I_{Vm}P_m(\eta _l) \bigg \}\omega ^*_l. \end{aligned}$$

To make the calculation simple we have made the substitution $$\eta _l=\big (\frac{t}{2}(\tau _l 
+1)\big )$$. Hence, the system of equations Eq. (9) will have ”$$5N+ 5$$” unknowns and 5*N* nonlinear algebraic equations. The five initial conditions are used to do for the answer:

10 $$\begin{aligned}&\sum _{m=0}^{N}S_{Hm}P_m(0)=k_1, \quad \sum _{m=0}^{N}I_{Hm}P_m(0)=k_2, \quad \sum _{m=0}^{N}R_{Hm}P_m(0)=k_3,\nonumber \\&\sum _{m=0}^{N}S_{Vm}P_m(0)=k_4,\quad \sum _{m=0}^{N}I_{Vm}P_m(0)=k_5. \end{aligned}$$

So Equations (9 and  10) yield a system containing $(5N+5)$ nonlinear algebraic equations and and the same number of unknowns $S_{Hm}, I_{Hm}, R_{Hm}, S_{Vm}, I_{Vm}$ for m=0,1,...,N. By substituting the unknown values into Eq. (8), a solution approximation for the stochastic SIR model may be produced.

## Basic reproduction number ($R_0$)

Equation (1) provide the dynamics of ZIKV disease,in which the two infectious classes are,

11 $$\begin{aligned} {\left\{ \begin{array}{ll} dI_H(t)=(1-\mu _1)\beta _1S_H(I_V+\vartheta I_H)-(\mu _H+\varsigma +\upsilon _1\mu _2)I_H,\\ dI_V(t)=(1-\mu _1)\beta _2S_VI_H-(\mu _V+\upsilon _2\mu _3)I_V.\\ \end{array}\right. } \end{aligned}$$

By employing the next-generation operator approach,the matrix F stands for new generated infections while the transition terms are taken in matrix V , thus we have

12 $$\begin{aligned} F = \begin{bmatrix} \frac{\vartheta \beta _1\Lambda _H}{\mu _H} &{} \frac{\beta _1\Lambda _H}{\mu _H} \\ \frac{\beta _2\Lambda _V}{\mu _V} &{}0 \\ \end{bmatrix}, V = \begin{bmatrix} \varsigma +\mu _H &{} 0 \\ 0 &{}\mu _V \\ \end{bmatrix}. \end{aligned}$$

The basic reproductive number, $R_0$ is given to us by the spectral radius of the matrix $FV^{-1}$, and hence:

$$\begin{aligned} R_0=\rho (FV^{-1})=\frac{\sqrt{\beta _1\Lambda _H(4 \varsigma \mu _H\beta _1\Lambda _V+\vartheta ^2\beta _1\mu _V^2\Lambda _H +4\Lambda _V\beta _2\mu _H^2)}+\Lambda _H\beta _1\mu _V \vartheta ^2}{2(\varsigma +\mu _H)\mu _V\mu _H}. \end{aligned}$$

## Stability and convergence of the spectral method

We first discuss the following stability, accuracy and convergence analysis for the proposed LSCM.

1. For stability analysis, we first investigate the eigenvalues of the linear operator $L$ in a matrix representation. For this, we have assume a differential equation in the form:

$$\begin{aligned} \frac{d}{dt} u(x, t) = Lu(x, t). \end{aligned}$$

To expanding $u(x, t)$ in terms of Legendre polynomials $P_n(x)$:

$$\begin{aligned} u_N(x, t) = \sum _{n=0}^{N} a_n(t) P_n(x). \end{aligned}$$

Apply the operator $L$ to $u_N$ is:

$$\begin{aligned} Lu_N(x, t) = \sum _{n=0}^{N} a_n(t) L P_n(x). \end{aligned}$$

Next we construct a matrix $A$ where:

$$\begin{aligned} A_{ij} = \int _{-1}^{1} P_i(x) L P_j(x) \, dx. \end{aligned}$$

For the stability analysis we requires the eigenvalues $\lambda _i$ of a matrix $A$ that satisfy:

$$\begin{aligned} \text {Re}(\lambda _i) < 0, \quad \forall \quad i. \end{aligned}$$

This confirm that the proposed system stable and also with time the errors do not grow exponentially that maintaining the stability of the numerical solution using LSCM.

2. Accuracy: The accuracy of the LSCM is due to its exponential convergence properties. Therefore, for a function $f(x)$ with an expansion:

$$\begin{aligned} f(x) \approx \sum _{n=0}^{\infty } a_n P_n(x), \end{aligned}$$

the error $E_N$ when approximating the given function $f$ with the first $N$ terms is:

$$\begin{aligned} E_N = \Vert f(x) - f_N(x) \Vert _{L^2} = \left( \int _{-1}^{1} \left| f(x) - \sum _{n=0}^{N} a_n P_n(x) \right| ^2 dx \right) ^{1/2}. \end{aligned}$$

For the sufficiently smooth function, coefficients $a_n$ exponentially decay that is:

$$\begin{aligned} |a_n| \le C e^{-\beta n}, \end{aligned}$$

where $C$ and $\beta$ are constants. This leads to an exponentially decreasing error:

$$\begin{aligned} E_N \le C e^{-\alpha N}, \end{aligned}$$

where $\alpha$ is related to smoothness of the function $f(x)$.

3. Convergence Analysis: Convergence of the LSCM is to determined by the decay of the proposed spectral coefficients. Therefore, for an analytic function $f(x)$:

$$\begin{aligned} f(x) \approx \sum _{n=0}^{\infty } a_n P_n(x), \end{aligned}$$

the coefficients $a_n$ decay as:

$$\begin{aligned} |a_n| \le C e^{-\gamma n}, \end{aligned}$$

where the parameter $\gamma$ totaly depends on a distance to the nearest singularity of $f$ in the complex plane. The given decay gives guarantee to the rapid convergence. Similarly, error bound derivation for a proposed function $f$ along with $m$ continuous derivatives, the error $E_N$ take the following form:

$$\begin{aligned} E_N = \Vert f(x) - f_N(x) \Vert _{L^2} = \left( \int _{-1}^{1} \left| f(x) - \sum _{n=0}^{N} a_n P_n(x) \right| ^2 dx \right) ^{1/2}. \end{aligned}$$

Now using the orthogonality of Parseval’s theorem and Legendre polynomials:

$$\begin{aligned} \Vert f(x) - f_N(x) \Vert _{L^2} = \left( \sum _{n=N+1}^{\infty } |a_n|^2 \right) ^{1/2} \end{aligned}$$

For an analytic function, $|a_n| \le C e^{-\beta n}$, so:

$$\begin{aligned} \Vert f(x) - f_N(x) \Vert _{L^2} \le \left( \sum _{n=N+1}^{\infty } (C e^{-\beta n})^2 \right) ^{1/2}. \end{aligned}$$

Evaluating the geometric series:

$$\begin{aligned}{} & {} \Vert f(x) - f_N(x) \Vert _{L^2} \le C e^{-\beta N} \left( \sum _{n=0}^{\infty } e^{-2\beta n} \right) ^{1/2} \\{} & {} \sum _{n=0}^{\infty } e^{-2\beta n} = \frac{1}{1 - e^{-2\beta }}. \end{aligned}$$

Thus:

$$\begin{aligned} \Vert f(x) - f_N(x) \Vert _{L^2} \le C' e^{-\beta N} \end{aligned},$$

where $C' = \frac{C}{(1 - e^{-2\beta })^{1/2}}$. This confirms that the exponential convergence of the proposed LSCM which highlighting its efficiency and accuracy.

As described in Eq. (2), we examine the stability analysis of the stochastic ZIKV model system in this section. Remember that the ”next generation approach” (NGM) states the following, as expounded above:

$$\begin{aligned} R_0=\rho (FV^{-1})=\frac{\sqrt{\beta _1\Lambda _H(4\varsigma \mu _H\beta _1\Lambda _V+\vartheta ^2\beta _1\mu _V^2\Lambda _H +4\Lambda _V\beta _2\mu _H^2)}+\Lambda _H\beta _1\mu _V\vartheta ^2}{2(\varsigma +\mu _H)\mu _V\mu _H} \end{aligned}.$$

### Theorem 1

The infected class defined in system Eq. (1) decreases inside the system and reaches an infectious-free equilibrium when $R_0\le 1,$ then ”$\big (S_{H}^{*}(t), I_{H}^{*}(t), R_{H}^{*}(t), S_V(t), I_{V}^{*}(t)\big )\rightarrow (\frac{\Lambda _H}{\mu _H}, 0, 0, \frac{\Lambda _V}{\mu _V+\upsilon _2\mu _H}, 0)$”. Moreover, if $R_0>1,$ then Eq. (1) has unique stable endemic equilibrium $E^{*}=\big \{S_{H}^{*}(t), I_{H}^{*}(t), R_{H}^{*}(t), S_{V}^{*}(t),I_{V}^{*}(t)\big \}$, is attained, where

$$\begin{aligned}&S_H^*(t)=\frac{(\mu _H+\varsigma +\upsilon _1\mu _2)I_V^*(t)}{\beta _1(1-\mu _1)(I_V^*(t)+\vartheta I_H^*(t))},\quad I_H^*(t)=\frac{\mu _H R_H^*(t)}{\varsigma +\upsilon _1\mu _2}, \quad R_H^*(t)=\frac{(\varsigma +\upsilon _1\mu _2) I_H^*(t)}{\mu _H},\\&S_V^*(t)=-\frac{(\mu _V+\upsilon _2 \mu _3) I_V^*(t)}{\beta _2I_H^*(t)(-1+\mu _1)}, \quad I_V^*(t)=-\frac{(-1+\mu _1)\beta _2S_V^*(t) I_H^*(t)}{\mu _V+\upsilon _2\mu _3}. \end{aligned}$$

### Proof

The system defined in Eq. (1) may be persuade by $S^*_H (t),I^*_H (t),R^*_H (t),S^*_V (t)$ and $I^*_V (t)$ The equations in the system may be solved if we think of E* as the endemic equilibrium of Eq. (1):

13 $$\begin{aligned}&\Lambda _H-{(1-\mu _1)\beta _1S^*_H(t)(I^*_H(t)_V+\vartheta I^*_H(t)) -\mu _HS^*_H(t)}=0,\nonumber \\&(1-\mu _1)\beta _1S^*_H(t)(I^*_V(t)+\vartheta I^*_H(t))-(\mu _H +\varsigma +\upsilon _1\mu _2)I^*_H(t)=0,\nonumber \\&(\varsigma +\upsilon _1\mu _2)I^*_H(t)-\mu _HR^*_H(t)=0,\nonumber \\&\Lambda _V-(1-\mu _1)\beta _2S^*_V(t)I^*_H-(\mu _V+\upsilon _2\mu _3)S^*_V(t)=0,\nonumber \\&(1-\mu _1)\beta _2S^*_V(t)I^*_H(t)-(\mu _V+\upsilon _2\mu _3)I^*_V(t)=0. \end{aligned}$$

The proof takes two scenarios into account as follow:

$$I^*_H(t)=0=I^*_V(t)$$ and $$I^*_H(t)>0$$,$$I^*_V(t)>0$$

First, for $$I^*_H(t)=0=I^*_V(t)$$,

While the fourth equation of the system produces $S^*_V (t)=\frac{\Lambda _V}{\mu _V+\upsilon _2\mu _3}$, the third equation of the proposed system given in Eq. (13) follows that $R^*_H(t) = 0$. The following is obtained by inserting these computed values into the system’s first equation: $S^*_H (t)=\frac{\Lambda _H}{\mu _H}$ At last, a state of equilibrium has been attained, ”$(S^*_H(t),I^*_H(t),R^*_H(t),S^*_V(t),I^*_V(t))\rightarrow (\frac{\Lambda _V}{\mu _V+\upsilon _2\mu _3},0,0,\frac{\Lambda _H}{\mu _H},0)$”. The relevant epidemic equilibrium is then found by using Maple-13 if $I^*_H(t)>0$,$I^*_V(t)>0$.

### Lemma 2

The entire region ${\mathbb {D}}$ remains a positive invariant set with in the context of the stochastic system provided in Eq. (2).

### Proof

As for our proposed model we have described the population as under $N(t)=N_V+N_H=S_H+I_H+R_H+S_V+I_V$ by Eqs. (2), we obtain

$$\begin{aligned} \frac{dN(t)}{dt}=\Lambda _H-\mu _HS_H+\Lambda _V-\mu _VN_V-\upsilon _2\mu _3N_V. \end{aligned}$$

Integrating and solving we have

$$\begin{aligned} N(t)&=N(0)e^{-\mu _Ht}+\frac{\Lambda _H}{\mu _H}+N(0)e^{-\mu _Vt} +\frac{\Lambda _V}{\mu _V} \\&\le \frac{\Lambda _H}{\mu _H}+\frac{\Lambda _V}{\mu _V}, \end{aligned}$$

providing that, the region is positively invariant.

### Lemma 3

The system defined in Eqs. (1) with variables $\big (S_H(t), I_H(t), R_H(t), S_V(t), I_V(t)\in {\mathbb {R}}^5\big )$ , for every initial condition, displays the following characteristics:

$$\begin{aligned} \mathop {\textrm{lim}}_{t\rightarrow \infty }\frac{\textrm{1}}{t} \int ^t_0{}\sigma _1 S\mathrm {(}s\mathrm {)}dW\mathrm {(}s\mathrm {)=0,} \end{aligned}$$

and similarly certainly for every class.

### Definition 4

Human infected individuals $I_H$ as well as the vector infected population $I_V$, become extinct if and only if $\mathop {\textrm{lim}}_{t\rightarrow \infty }I(t)=0$ in Eq. (2).

### Theorem 5

If max$\bigg \{\big (\frac{\beta _1\mu _H}{\Lambda _H}+\frac{\beta _2\mu _V}{\Lambda _V}\big ),\big (\frac{\beta ^{2}_H}{2(\mu _H+\varsigma +\upsilon _1\mu _2)}+\frac{\beta ^{2}_V}{2\mu _V}\big )\bigg \}<\sigma ^{2}$ or $\big (\frac{\beta _1\mu _H}{\Lambda _H}+\frac{\beta _2\mu _V}{\Lambda _H}\big )>\sigma ^{2}$ with ${{\overline{R}}}_0\mathrm {<}1$, then the stochastic systems represented by Eq. (2) exponentially approaches to zero. Consequently for ${{\overline{R}}}_0\mathrm {>}1$ the infected class does not vanish and remain present in Eq. (2),where ${{\overline{R}}}_0\mathrm {=}R_0-\big (\frac{\sigma ^2\Lambda ^2_H}{2\mu ^2_H(\mu _H+\varsigma +\upsilon _1\mu _2)}+\frac{\sigma ^2\Lambda ^2_V}{2\mu ^3_V}\big )$

### Proof

Assume for the moment that the initial conditions of the stochastic system given by Eq. (2) are true and that a solution , $S_H(t),I_H(t),R_H(t),S_V(t),I_V(t)$ exists. that satisfies these conditions. Further $I=I_H+I_V$ The following form might then be obtained using the Itô’s formula:

14 $$\begin{aligned} d\ln I(t)&=\bigg (\beta _1 S_H-(\mu _H+\varsigma +\upsilon _1\mu _2)-\frac{\sigma ^2S^2_H}{2}\bigg )dt+\sigma S_HdW(t)+\bigg (\beta _2S_V-\mu _V-\frac{\sigma ^2S^2_V}{2}\bigg )dt\nonumber \\&+\sigma S_VdW(t). \end{aligned}$$

Eq. (14) need to be integrated with limits from 0 to *t* taking the following shape:

15 $$\begin{aligned} \ln I(t)&=\ln I_H(0)+\int _{0}^{t}\bigg (\beta _1 S_H-(\mu _H+\varsigma +\upsilon _1\mu _2)-\frac{\sigma ^2S^2_H}{2}\bigg )dt+\int _{0}^{t}\sigma S_HdW(t)\nonumber \\ {}&+\ln I_V(0)+\int _{0}^{t}\bigg (\beta _2S_V-\mu _V-\frac{\sigma ^2S^2_V}{2}\bigg )+\int _{0}^{t}\sigma S_V(t)dW(t). \end{aligned}$$

We discuss two cases, if $\sigma ^2>\big (\frac{\beta _1\mu _H}{\Lambda _H}+\frac{\beta _2\mu _V}{\Lambda _H}\big )$, then:

16 $$\begin{aligned} \ln I(t)&\le \ln I_H(0)+\bigg (\frac{\beta ^2_H}{\sigma ^2} -(\mu _H+\varsigma +\upsilon _1\mu _2)\bigg )t+\int _{0}^{t}\sigma S_HdW(t)\nonumber \\ {}&+\ln I_V(0)+\bigg (\frac{\beta ^2_V}{\sigma ^2}-\mu _V\bigg )t+\int _{0}^{t}\sigma S_V(t)dW(t). \end{aligned}$$

Divide Eq. (16) by a positive *t*, then

17 $$\begin{aligned} \frac{1}{t}\ln I(t)&\le \frac{1}{t}\ln I_H(0)+\bigg (\frac{\beta ^2_H}{\sigma ^2}-(\mu _H+\varsigma +\upsilon _1\mu _2) \bigg )+\frac{1}{t}\int _{0}^{t}\sigma S_HdW(t)\nonumber \\ {}&+\frac{1}{t}\ln I_V(0)+\bigg (\frac{\beta ^2_V}{\sigma ^2}-\mu _V\bigg )+\frac{1}{t}\int _{0}^{t}\sigma S_V(t)dW(t). \end{aligned}$$

With the application of Lemma 5.4, and $\lim _{t\rightarrow \infty }$ the following result is obtained from Eq. (17):

$$\begin{aligned} \lim _{t\rightarrow \infty }{\frac{\ln I(t)}{t}}\le -\bigg ((\mu _H+\varsigma +\upsilon _1\mu _2)-\frac{\beta ^2_H}{\sigma ^2}\bigg )-\bigg (\mu _V-\frac{\beta ^2_v}{\sigma ^2}\bigg )<0. \end{aligned}$$

Therefore,if $\sigma ^2>\bigg (\frac{\beta ^2_H}{2(\mu _H+\varsigma +\upsilon _1\mu _2)} +\frac{\beta ^2_V}{2\mu _V}\bigg )$ Which show that $\lim _{t\rightarrow \infty }I(t)=0.$

The second case if $\sigma ^2<\bigg (\frac{\beta _1\mu _H}{\Lambda _H}+\frac{\beta _2\mu _H}{\Lambda _V}\bigg )$, then from Eq. (15) we have

18 $$\begin{aligned} \ln I(t)\le&\ln I_H(0)+\bigg (\frac{\beta _1\Lambda _H }{\mu _H}-\frac{\sigma ^2_H\Lambda ^2_H}{\mu ^2_H}-(\mu _H+\varsigma +\upsilon _1 \mu _2)\bigg )t+\int _{0}^{t}\sigma S_H(t)dW(t)\nonumber \\ +&\ln I_V(0)+\bigg (\frac{\beta _2\Lambda _V }{\mu _V}-\frac{\sigma ^2_V\Lambda ^2_V}{\mu ^2_V}-(\mu _V+\varsigma +\upsilon _2\mu _2)\bigg )t+\int _{0}^{t}\sigma S_V(t)dW(t). \end{aligned}$$

The following equation is the result of division of Eq. (18)’s both sides by positive *t* i.e:

19 $$\begin{aligned} \frac{1}{t}\ln I(t)&\le \frac{1}{t}\ln I_H(0)+(\mu _H+\varsigma +\upsilon _1\mu _2)\bigg (\frac{\beta _1\Lambda _H }{\mu _H(\mu _H+\varsigma +\upsilon _1\mu _2)}-\frac{\sigma ^2\Lambda _H^2}{\mu _H^2(\mu _H +\varsigma +\upsilon _1\mu _2)}-1\bigg )\nonumber \\&+\frac{1}{t}\int _{0}^{t}\sigma S_H(t)dW(t)+\frac{1}{t}\ln I_V(0)+(\mu _V)\bigg (\frac{\beta _1\Lambda _H }{\mu _V^2}-\frac{\sigma ^2\Lambda _H^2}{\mu _V^3}-1\bigg )+\frac{1}{t}\int _{0}^{t}\sigma S_V(t)dW(t). \end{aligned}$$

Again with the application of Lemma 5.4, and $\lim _{t\rightarrow \infty }$ the following result is obtained from Eq. (19):

$$\begin{aligned} \lim _{t\rightarrow \infty }{\frac{\ln I(t)}{t}}\le \mu _V(\mu _H+\varsigma +\upsilon _1\mu _2)\big )({\bar{R}}_0-1). \end{aligned}$$

For $${\bar{R}}_0<1$$,

$$\begin{aligned} \lim _{t\rightarrow \infty }{\frac{\ln I(t)}{t}}<0. \end{aligned}$$

This shows that $\lim _{t\rightarrow \infty }I(t)=0.$

### Theorem 6

Equation (2) indicates the existence of the contaminating population, $I_H$ and $I_V$, if the reproduction number ${\bar{R}}_0$ is greater than 1. For evidence, see^39^.

## Numerical discussions

**Figure 1:**
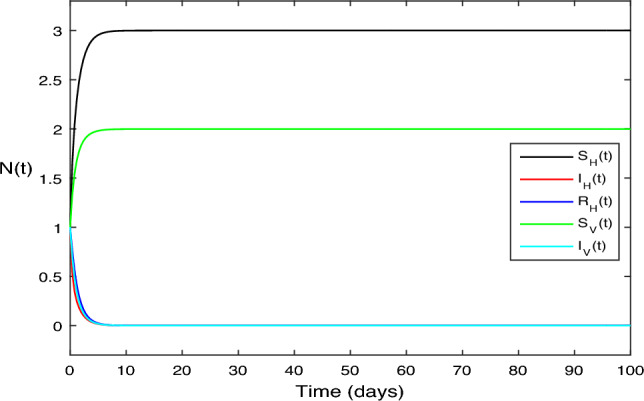
The solution behavior of deterministic system (Eq. 1), for $R_0<1$.

The numerical test problems are included in this section. Both the stochastic system Eq. (2) and the deterministic system Eq. (1) obtain and describe the numerical findings. The numerical results for the specified models are found using the Legendre spectral collocation method.The outcomes are displayed in Fig. 1 through 10. On a home computer, related computations are performed using the programs Matlab and Maple.

In Fig. 1, it was simple to set the right parameter values. We used the following parameter values in the context of the deterministic system given by Eq. (1): $\beta _1 = 0.1, \beta _2 = 0.2,\vartheta =0.3, \mu _H = 1, \mu _V = 1, \upsilon _1 = 0.7, \upsilon _2 = 2, \varsigma = 3, \mu _1 = 0.1, \mu _2 = 0.1, \mu _3 = 0.01$. With these parameter values, the calculation produced a reproduction number $R_0 = 0.813587 < 1$. In this case, the application of Theorem 1, suggest that disease die out and hence we have only $S_H$ and $S_V$ classes refer to as $E_0(3, 0, 0,2,0)$, where the vector population $\Lambda _H = 3$ and the human population $\Lambda _V = 2$.

**Figure 2:**
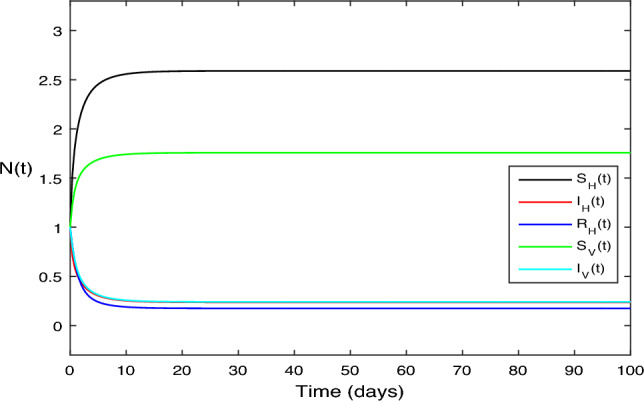
The solution behavior of deterministic system (Eq. 1), for $R_0>1$.

Similarly, in Fig. 2 we increase the contact parameters $\beta _1 = 0.75$ and $\beta _2 = 0.75,$” and with all other parameter values consistent with those shown in Fig. 1. The reproduction number in this case is $R_0=1.675 > 1$, and hence disease exist in the population as Fig. 2 illustrates. These results are consistent with Theorem 1 claims.

**Figure 3:**
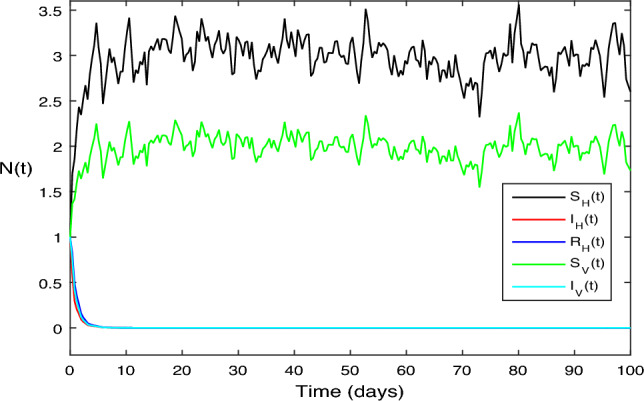
The solution behavior of stochastic ZIKV system (Eq. 2),when $\bar{R_0}<1$.

In Fig. 3, for the stochastic system Eq. (2), we selected parameter values $\beta _1 = 0.1, \beta _2=0.2,\vartheta =0.3,\mu _H = 1, \mu _V = 1,\upsilon _1 = 0.7, \upsilon _1 = 2,\varsigma = 3. \mu _1 = 0.05, \mu _2 = 0.05,\mu _3 = 0.2$. A quick calculation reveals the situation, $\sigma ^{2}<\max \bigg \{\big (\frac{\beta _1\mu _H}{\Lambda _H}+\frac{\beta _2\mu _V}{\Lambda _H}\big ),\big (\frac{\beta ^{2}_H}{2(\mu _H+\varsigma +\upsilon _1\mu _2)}+\frac{\beta ^{2}_V}{2\mu _V}\big )\bigg \}<\sigma ^{2}$ Moreover, Eq. (2) predicts a decrease in the number of infected persons in the vector and human populations inside the system, in accordance with the ideas presented in Theorem 5, with $\bar{R_0} = 0.6534 < 1$. Figure 3 provides a clear illustration of this pattern.

**Figure 4:**
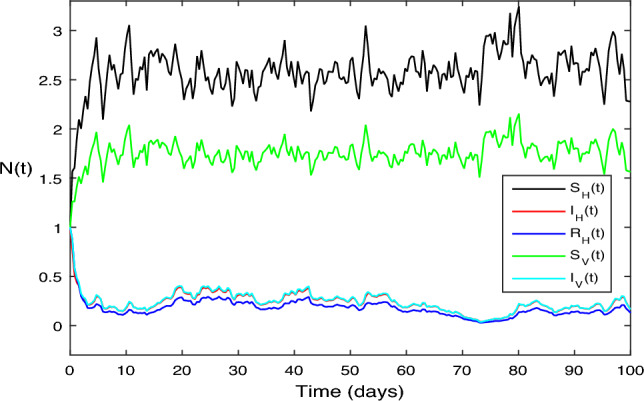
The solution behavior of stochastic ZIKV system (Eq. 1), for $\bar{R_0}>1$.

Also in Fig. 4, a simple calculation shows that the deterministic system given by Eq. (2) meets the requirement when we select $\beta _1 = 0.75$ and $\beta _2 = 0.75$ while leaving all other parameter values constant. This scenario is similar to the one shown in Fig. 3.

$\sigma ^{2}<\max \bigg \{\big (\frac{\beta _1\mu _H}{\Lambda _H} +\frac{\beta _2\mu _V}{\Lambda _H}\big ),\big (\frac{\beta ^{2}_H}{2(\mu _H+\varsigma +\upsilon _1\mu _2)} +\frac{\beta ^{2}_V}{2\mu _V}\big )\bigg \}<\sigma ^{2}$ and $\bar{R_0}= 1.5 > 1$, Accordingly, Theorem 5 states that the infected individuals in the vector and human populations are, in fact, present in the system given by Eq. (2); Fig. 4 illustrates this reality.

**Figure 5:**
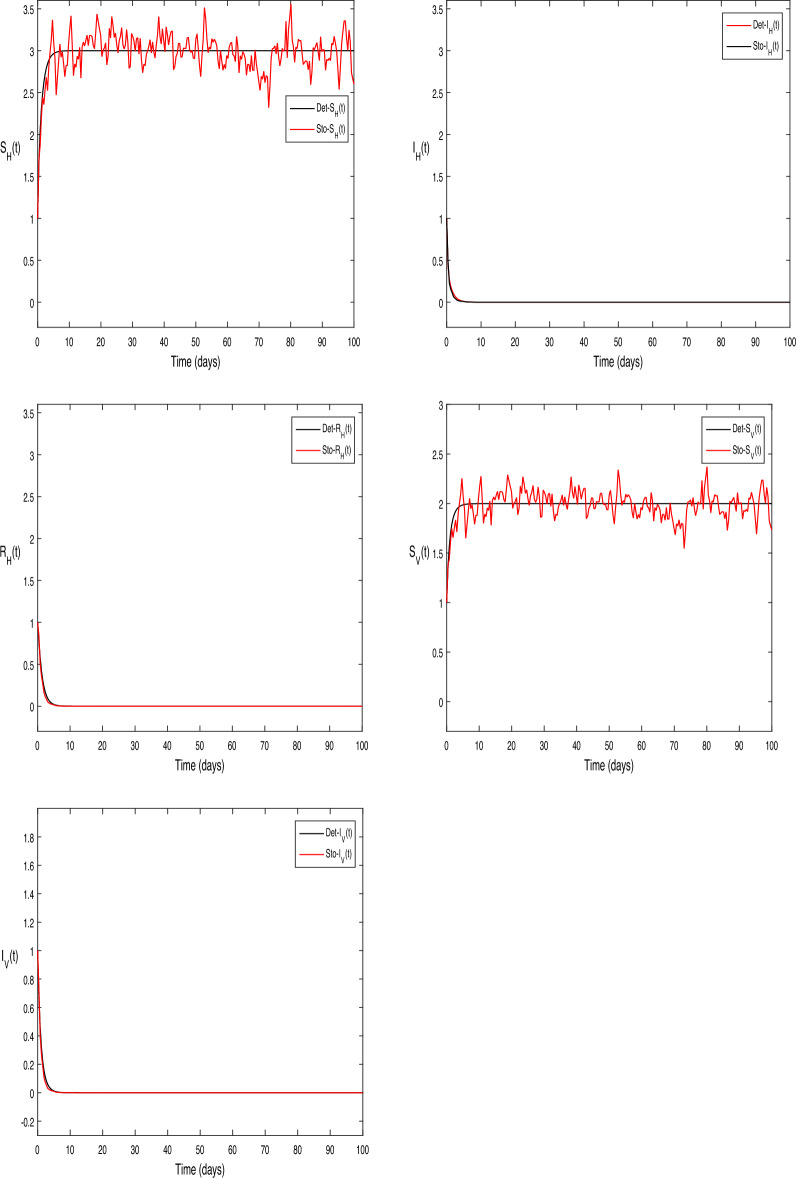
Since the deterministic equations (1) are more precise, we will now compare them to the stochastic $R_0<1, \bar{R_0}<1$.

The Fig. 5, graph shows a comparison between the stochastic and deterministic human systems with the parameter values set as follows: $\beta _1 = 0.1, \beta _2 = 0.2, \mu _H = 1, \mu _V = 1, \upsilon _1 = 0.7, \upsilon _2 = 2, \varsigma = 3, \mu _1 = 0.05, \mu _2 = 0.05, \mu _3 = 0.2.$ When these parameter values are applied, a simple calculation shows that in the deterministic system. Eq. (1) $R_0=0.75<1$ and Eq. (2)$\sigma ^{2}<\max \bigg \{\big (\frac{\beta _1\mu _H}{\Lambda _H} +\frac{\beta _2\mu _V}{\Lambda _H}\big ),\big (\frac{\beta ^{2}_H}{2(\mu _H+\varsigma +\upsilon _1\mu _2)} +\frac{\beta ^{2}_V}{2\mu _V}\big )\bigg \}<\sigma ^{2}$and $\bar{R_0}= 0.25 < 1$.

**Figure 6:**
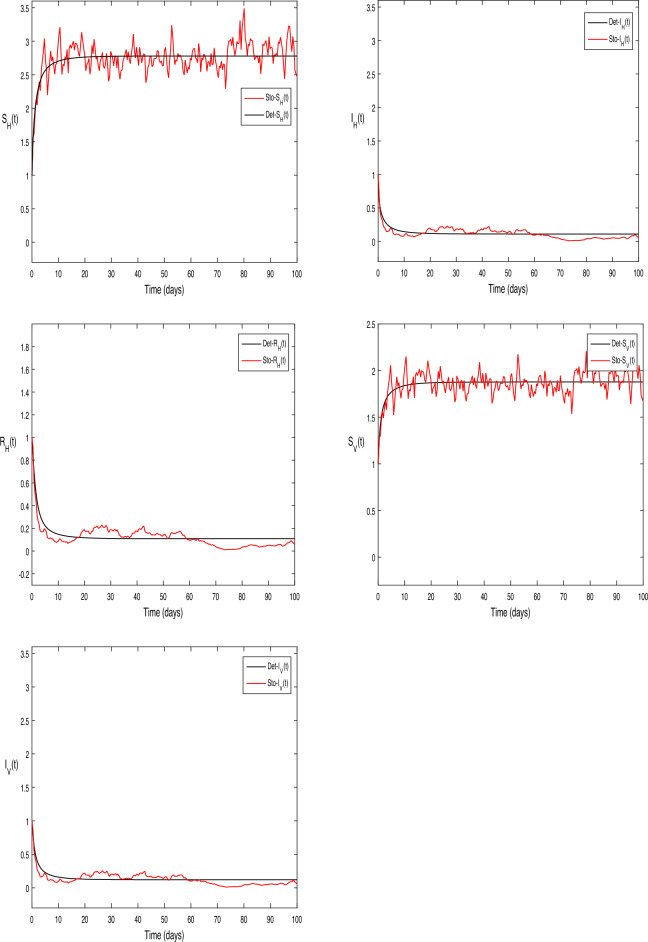
Ratio of stochastic and deterministic system for each class with both $R_0>1, \bar{R_0}>1$.

In Fig. 6, we assigning the following value to parameters, to compared the behavior of deterministic and stochastic model for both humans and vectors separately in this figure where, $\beta _1 = 0.1, \beta _2 = 0.2,\vartheta =0.3, \mu _H = 1, \mu _V = 1, \upsilon _1 = 0.7, \upsilon _2 = 2, \varsigma = 3, \mu _1 = 0.1, \mu _2 = 0.1, \mu _3 = 0.01$. Calculation in this case yields $R_0 = 1.675 > 1$ and $\bar{R_0}= 1.43 < 1$.

**Figure 7:**
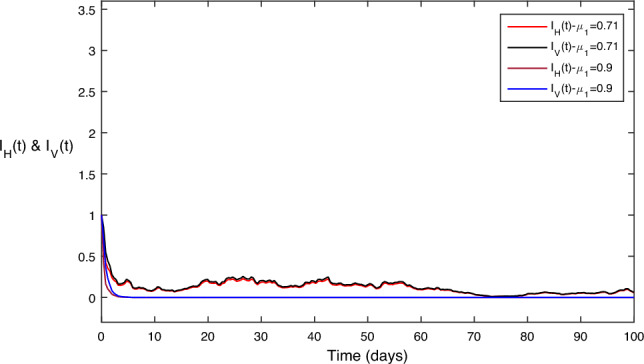
Different values for the prevention control parameter $\mu _1$; as we increase then both the disease classes goes to zero.

In Fig. 7, the comparative impact of prevention control parameter on disease transmission can be observed in Fig. 7. We can clearly see from the comparison that disease approaches almost zero for the maximum value of while keeping other values constant.

**Figure 8:**
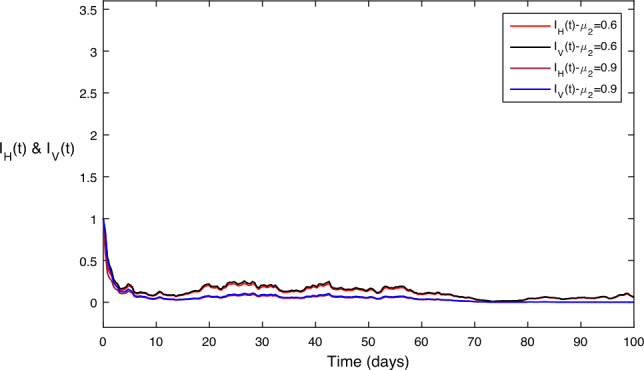
Different values for the treatment control parameter $\mu _2$; as we increase then both the disease classes goes to zero.

Figure 8 shows the impact of treatment control parameter on disease transmission. This can be observed that has comparatively lesser effect on disease transmission for its maximum value. While keeping other values constant.

**Figure 9:**
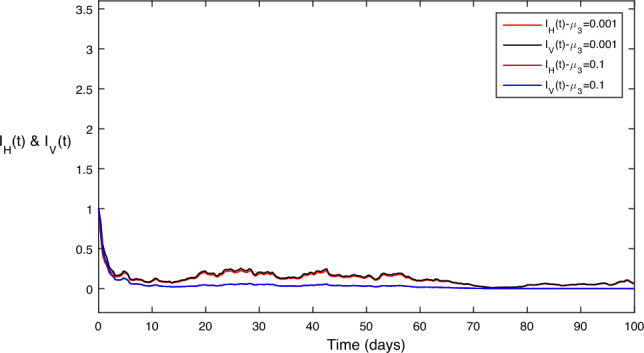
Different values for the treatment pesticide control parameter $\mu _3$; as we increase then both the disease classes goes to zero.

In Fig. 9, we compare the impact of insecticide control parameter on disease transmission for its maximum and minimum values. The fact can be cleanly observed that maximizing has a greater impact on disease control by changing the value of from minimum to maximum.

**Figure 10:**
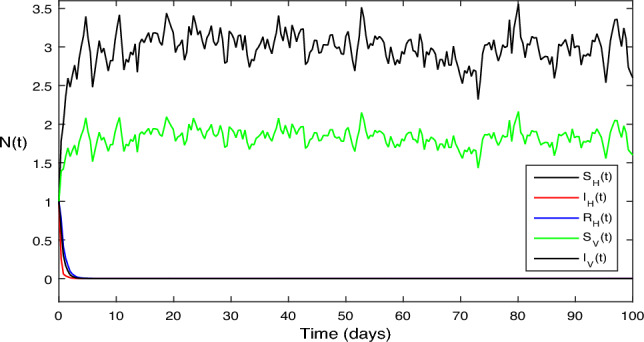
Using maximum values of all the three control parameters.

In Fig. 10, the following parameter values, the effect of maximum values of control parameters are shown in Fig. 10: $\beta _1 = 0.1, \beta _2 = 0.2,\vartheta =0.3, \mu _H = 1, \mu _V = 1, \upsilon _1 = 0.7, \upsilon _2 = 2, \varsigma = 3, \mu _1 = 0.1, \mu _2 = 0.1, \mu _3 = 0.01$. With the combine application of all control parameters, disease vanishes quickly.

### Ethics approval

Our study did not require an ethical board approval because it did not contain human or animal trials.

## Conclusion

The computational investigation of the stochastic ZIKV optimal control model using the LSCM determined several key vitality. The stability, convergence and accuracy properties of the proposed LSCM make it a vigorous choice for solving such complex differential equations inherent in a epidemiological systems. The stability analysis confirms that given LSCM maintains the probity of the approximate solution over time by ensuring that the errors do not grow up exponentially. Similarly the convergence analysis further highlights the efficacy of the proposed method which shows the spectral coefficients that rapidly decay, ensuring the reliable convergence to the true solution. However, the accuracy of the LSCM is evident through its exponential convergence which grant for efficient approximation of functions with having minimal computational effort.

Moreover, to incorporating the random interference into the dynamical model through white noise improve the reality of the simulations that the stochastic nature reflecting the disease transmission. The choice of parameter values, totaly based on a epidemiological data and realistic biological phenomena that ensures the simulations are both meaningful and relevant. The given approach provides beneficial understanding into the optimal control strategies for executing the spread of the ZIKV which show the practical applications of advanced numerical techniques in epidemiology and public health.

Overall, the LSCM proves to be a powerful tool for the computational modeling such as stochastic epidemiological models which offering the high stability, convergence rates and accuracy. The LSCM application to the ZIKV model underscores its potential in advantageous the development of effective control strategies of infectious diseases.

Future research could also explore the application of LSCM to investigate the impact of different types of random perturbations on the stability, to other stochastic epidemiological models and control strategies of infectious diseases.

## Data Availability

The datasets used and/or analysed during the current study available from the corresponding author on reasonable request.
